# Factors Related to Cigarette Smoking Initiation and Use among College Students

**DOI:** 10.1186/1617-9625-3-1-27

**Published:** 2005-12-15

**Authors:** Diane Von Ah, Sheryl Ebert, Anchalee Ngamvitroj, Najin Park, Duck-Hee Kang

**Affiliations:** 1 Indiana University, School of Nursing, Indianapolis, Indiana, USA; 2 Department of Psychology, University of Alabama at Birmingham, USA; 3 College of Nursing, The Thai Red Cross Society, Bangkok, Thailand; 4 School of Nursing, University of Alabama at Birmingham, USA

## Abstract

The purpose of this cross-sectional study was to examine the impact of personality factors (neuroticism, extraversion, openness, agreeableness, and conscientiousness), cognitive factors (sense of coherence and self-efficacy), coping resources (family and friend social support) and demographic factors (gender and ethnicity) on cigarette smoking behaviors (initiation, frequency, and amount of cigarette smoking) among college students. A total of 161 U.S. college students, aged 18–26, who enrolled in an introductory psychology course completed self-report questionnaires. The majority of the students had tried smoking (55%); among those who had tried, 42% were current smokers. The majority (77%) who had smoked a whole cigarette did so at age 16 years or younger. Students who reported lower levels of conscientiousness and self-efficacy had a greater likelihood to had tried cigarette smoking. Also, students who had lower levels of self-efficacy reported smoking more frequently and greater quantities of cigarettes than students with higher levels of self-efficacy. Self-efficacy was the most significant predictor of smoking behaviors. Health promotion programs focused on self-efficacy may be an effective tool for reducing the initiation, frequency, and amount of cigarette smoking among college students.

## Introduction

Cigarette smoking is the leading cause of preventable death in the United States (U.S.)[1]. Although cigarette smoking among adults has steadily declined over the past decade, smoking among college students has risen sharply [2]. In the U.S, it is estimated that approximately 29% of those, 18 to 24 years of age, smoke [3]. Similarly, Steptoe & Wardle (2001) reported that 22.9% and 19.8% of Western and Eastern European university students were regular smokers [4]. Coupled with this increase in smoking is the concern that younger smokers, such as college students, do not heed smoking-associated health warnings. Kvis and colleagues (1995) found that younger smokers (18–29 years of age) are less concerned about health outcomes associated with smoking than older adults [5]. Other researchers have reported that smoking prevalence in college students is complicated by the fact that these young adults believe that they can easily quit smoking [6], ignoring its addictive properties, and ultimately believe they can be spared from the long-term effects of smoking [7]. Elucidating determinants of cigarette smoking behaviors among college students, thus, would aid healthcare professionals to target intervention programs to those most in need.

## Background

Individual personality factors, cognitive factors, and coping resources may play a key role in determining which college students will have a propensity to initiate and continue to smoke. Personality factors as stable and distinctive traits of an individual may account for variability in health perceptions [8]. The proposition of the Five Factor Model of Personality is that people have consistent and enduring individual differences based on their personality. Personality factors include neuroticism (e.g., nervous or high-strung), extraversion (e.g., energetic or outgoing), openness (e.g., original or creative), agreeableness (e.g., accommodating or obliging), and conscientiousness (e.g., careful or incorruptible) [9,10]. Researchers have shown that neuroticism is associated with smoking onset in young people [11-13] and continued cigarette smoking in adults [14]. Individuals with high neuroticism tend to be impulsive and anxious, and are less likely to adhere to positive health behaviors even when the benefits are known [15]. Smokers and regular alcohol drinkers scored higher on extraversion than nonsmokers and nondrinkers [16]. Higher conscientiousness, on the other hand, was associated with protective health behaviors, such as regular exercise [15]. Although personality factors have been examined individually on health behaviors, few studies have comprehensively examined the associations between personality factors and cigarette smoking. All five major personality factors, thus, were examined in association with smoking behaviors among college students.

Cognitive factors, such as sense of coherence and self-efficacy, may also play an important role in determining smoking behaviors. Sense of coherence is a global orientation to life that reflects the degree to which a person feels confident that life is understandable, manageable, and meaningful [17,18]. Individuals with a high sense of coherence are believed to be better equipped at mobilizing the necessary resources to meet life demands. Individuals with high levels of sense of coherence are more likely to engage in positive health behaviors, such as regular exercise [19]. Conversely, Van Loon et al. (2001) found that women who smoked reported lower levels of sense of coherence than those who had never smoked[20]. These findings suggest that sense of coherence may play a significant role in smoking behaviors. However, this relationship has not been examined among college students.

Self-efficacy is well known to influence health behaviors [21]. Bandura's Theory of Self-Efficacy (1977) suggests that behavior is best predicted by an individual's confidence in their ability to accomplish a given task. Self-efficacy may impact health by influencing the adoption of health promoting behaviors, cessation of unhealthy behaviors, and/or the maintenance of behavioral changes when faced with difficult situations [22]. Kear (2002) found that self-efficacy to resist cigarette smoking was a significant determinant of smoking behavior. Similarly, Kvis and colleagues (1995) found that increased smoking self-efficacy is an important predictor for quitting smoking among 18–29 year olds. The role of self-efficacy on smoking, however, needs to be further examined along with other personality and cognitive factors among college students.

Social support, a coping resource, has been shown to positively influence health [23,24]. Previous research has generally indicated that adults with high levels of social support are less likely to engage in substance use [25-27]. Conversely, students with a negative social support network are especially at risk to develop poor health behaviors. College students with low levels of overall social support engaged in risky health behaviors including substance use of cigarettes and alcohol, clearly suggesting a potentially important role of social support on choosing healthy lifestyles [28]. Empirical findings, however, have been mixed. In general, parental emotional social support is believed to act as a protective factor and lower the likelihood of substance use [29,30]. Teenagers are less likely to smoke when parents are involved in their children's activities [31] and are supportive [32]. Similarly, parental emotional support was inversely related to tobacco, alcohol, and marijuana use among adolescents. Lack of family support, on the other hand, was a significant barrier to smoking cessation among Australian teenagers [33]. These findings suggest that family social support has a positive influence on health promoting behaviors. In comparison, friend or peer social support has been linked as a primary factor for adolescents to initiate cigarette smoking [34,35] and reduce their attempts to quit smoking [36].

Adolescents with friends who smoke are more likely to initiate smoking than those with friends who do not smoke [34]. Further research is needed to examine the varying role of social support from family and friends on smoking behaviors in college students many of who are away from home for the first time.

Demographic factors, such as gender and ethnicity, may also impact health behaviors [37]. Females are more likely than males to practice protective health behaviors [38], whereas male gender is a significant predictor of smoking initiation among adolescents [39,40]. Although ethnicity may also be an important factor in smoking behaviors, the majority of studies have been conducted with White subjects [37]. Kann (1993) found that White adolescents were more likely to smoke cigarettes than Non-white adolescents [41]. In general, however, little is known about the impact of gender and ethnicity on smoking behaviors, particularly among college-aged students [42].

In summary, empirical research has been limited in that it has failed to simultaneously address the aforementioned determinants on smoking behaviors among college-age students. The purpose of this study, therefore, was to examine the impact of the five major personality factors, sense of coherence, smoking self-efficacy, family and friend emotional social support, gender and ethnicity on smoking behaviors among college students.

## Method

This study is part of a larger study in which the impact of various psychosocial factors was examined on a number of select health behaviors among college students [21]. In this study, we focused on specific cigarette smoking behaviors including the number of lifetime smokers [43], smoking initiation, frequency, and amount of cigarette smoking, rather than overall use of tobacco. Based on the CDC guidelines, a lifetime smoker is defined as an individual who has ever tried smoking, even one or two puffs; smoking initiation was defined as the age at which an individual first smoked a whole cigarette; and a current smoker was defined as an individual who smoked a whole cigarette within the last 30 days [43].

### Participants

Participants consisted of 161 undergraduate students enrolled in introductory psychology courses at a Southern University in the U.S. Students were recruited by announcing the purpose and nature of the study in class during the semester and posting the schedule of data collection dates. Research team members were available to answer any questions during recruitment and data collection. As an incentive to participate in the study, students received two extra credit research points that was approved by the Department of Psychology of the University. The introductory psychology course had approximately 400 students enrolled at mid-semester, and thus, the overall response rate for the study was 40%. The study protocol was approved by the Institutional Review Board and consent was obtained from participants prior to data collection.

#### Instruments

The NEO Five Factor Inventory (NEO-FFI^©^) [9,10] is a 60-item personality inventory that was designed to measure five personality factors: Neuroticism, Extraversion, Openness, Agreeableness, and Conscientiousness. Responses on the NEO-FFI ranged from Strongly Disagree = 0 to Strongly Agree = 4. Twenty-seven items were reverse scored, following the scoring instructions. Each personality factor had 12 items with a score range of 0–48, higher scores indicating a greater impact of that personality factor. The NEO-FFI scales showed correlations of .75 to .89 with the longer version, the NEO Personality Inventory [9]. Construct validity of responses has been shown relative to other measures such as the California Psychological Inventory while divergent validity of responses has been demonstrated vis-à-vis psychopathology scales (e.g., Millon Clinical Multiaxial Inventory)[9]. Chronbach's coefficient alphas for five subscales were .83, .76, .69, .70, and .74 in our sample.

Sense of coherence (SOC) was assessed by a 29-item self-report instrument on which participants were asked to respond on a 7-point Likert-scale [18] to questions with opposing anchors (e.g., life has had no clear goals or purpose versus very clear goals and purpose). The SOC contains three subscales: comprehensibility, manageability, and meaningfulness [17,18]. The total score, which ranges from 29 (low SOC) to 203 (high SOC), was used in this study. The SOC scale is a reliable, valid, and cross cultural instrument [44] and has been used previously with college students [8]. Cronbach's coefficient alpha was .86 for the total scale in this study.

Smoking self-efficacy was measured by one item of the 4-item tobacco self-efficacy questionnaire utilized in our previous study [21]. Based on Bandura's Theory of Self-Efficacy [45], smoking self-efficacy was measured to indicate the respondent's confidence specifically in their resistance to smoking on a scale of 0 to 10. A higher score was indicative of a higher level of smoking self-efficacy. The original 4-item tobacco self-efficacy questionnaire had Chronbach's coefficient alpha of .90.

The Norbeck Social Support Questionnaire (NSSQ) was utilized to determine the type (emotional support) and source of social support (family and friends) [46,47]. The NSSQ has nine questions, and subjects were asked to list up to 24 significant others in their life and then, rate the level of support they perceived to receive from them on a Likert scale ranging from 0 (not at all) to 4 (a great deal). The particular type of emotional social support for this study was measured by summing their response to four questions, two affect and two affirmation questions from only family and friends. Aid or instrumental support could be calculated (two questions) but was not used in this study due to its high correlation with emotional support (r = .95). Concurrent validity estimates range from .24–.41, indicating moderate evidence of construct validity [47]. Chronbach's coefficient alphas were .95, .95, and .94 in our sample for the total scale and family and friend emotional support subscales, respectively.

Demographic information of age, gender, and ethnicity was collected using a questionnaire. For ethnicity, participants were asked to mark one of the five categories: American Indian or Alaskan Native, Asian, Black or African American, Native Hawaiian or Other Pacific Islander, or White. In analyzing the data, ethnicity was collapsed into white versus non-white, because there were few Asian or Hispanic participants.

Smoking behaviors, including lifetime smoker, smoking initiation, and the frequency and amount of cigarette smoking, were measured with questions refined from the Behavioral Risk Factor Surveillance System [48] and the Youth Risk Behavior Surveillance System [49]. The number of lifetime smokers was assessed with a dichotomous question asking participants if they had "ever tried cigarette smoking, even one or two puffs." Smoking initiation was assessed by asking at what age participants had first smoked a whole cigarette. The last two questions sought information on the participant's frequency of smoking in the last 3 months from never to every day and the amount of their smoking (number of cigarettes smoked per day). Chronbach's coefficient alpha was .98 for the 4-item cigarette smoking behavior questionnaire in this study.

## Data analysis

Logistic regression was used to examine the direct effects of personality factors (neuroticism, extraversion, openness, agreeableness, and conscientiousness), cognitive factors (sense of coherence and smoking self-efficacy), coping resources (family and friend emotional social support) and demographic variables (gender and ethnicity) on cigarette smoking initiation. Multiple linear regression was used to determine the contribution of the predictor variables on cigarette smoking frequency and quantity. The study variables were found to have normal distributions and only weak to moderate correlations and therefore met the assumptions for the analyses used [50].

## Results

### Participant Characteristics

The majority of the 161 participants were females (73%). The mean age was 19.7 (SD = 4.09) years with a range from 18 to 26 years. The sample was distributed between White (44%) and Non-white (56%) respondents. The overwhelming majority of participants reported they were single (91%), with 7% reporting being married, and another 2% divorced.

### Descriptive Statistics

**Figure 1:**
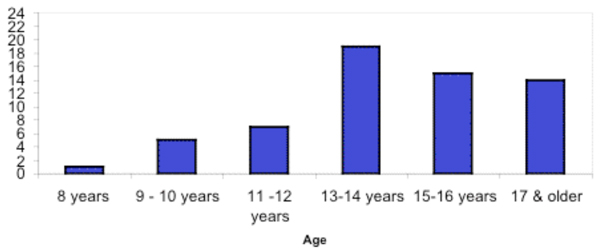
**Number of students and the age of first initation of smoking**.

Over half of the participants (88 out of 161 or 55%) reported having ever tried cigarette smoking (had at least one or two puffs), 42% of which were current smokers. Figure 1 displays the number of students who smoked a whole cigarette and the age at which they first initiated smoking. The majority of students (77%) who had smoked a whole cigarette did so at 16 years of age or younger. In regards to gender, there was not a significant difference between the number of males and females who had tried smoking, p = 0.60. Twenty-five out of the 43 males (58%) and 63 out of 118 females (53%) had tried cigarettes. However, there was a significant difference in the total number of White versus Non-white participants who reported having tried smoking, p = 0.02. Forty-six out of 70 White participants (66%) and 42 out of 90 Non-white participants (47%) had tried smoking cigarettes. As can be seen in Table 1, the students reported moderate levels of the personality factors with conscientiousness being slightly higher than the other resistant factors. Sense of coherence was moderate (125.5 ± 18.2), while self-efficacy was quite high (9.06 ± 2.42). The levels of family emotional support (58.1 ± 38.7) were ranked slightly higher than the levels of friend emotional support (51.5 ± 50.5).

**Table 1:** Descriptive statistics of Personality, Sense of Coherence, Smoking Self-efficacy, and Social Support.

		Possible Range	Mean (SD)
Personality Factors	Neuroticism	0–48	22.7 (8.1)
	Extraversion	0–48	30.3 (6.5)
	Openness	0–48	26.2 (5.7)
	Agreeableness	0–48	30.3 (5.7)
	Conscientiousness	0–48	32.1 (5.7)
			
Sense of coherence	Total	29–203	125.5 (18.2)
Self-efficacy	Smoking Self-efficacy	0–10	9.06 (2.42)
Family Social Support	Emotional	0–384	58.1 (38.7)
Friend Social Support	Emotional	0–384	51.5 (50.5)

### Smoking Initiation

Although slightly more males reported having tried cigarette smoking than females, gender was not a significant predictor of cigarette smoking initiation. In regards to ethnicity, individuals identified as non-white were less likely to smoke than their white counterparts, although this trend was not statistically significant, p = 0.06.

As shown in Table 2, the majority of personality and cognitive factors and coping resources examined in this study did not have a significant impact on smoking initiation. Only conscientiousness and self-efficacy showed a significant impact on smoking initiation. Students with higher levels of conscientiousness and self-efficacy were less likely to have tried cigarette smoking, OR = 0.87, p = 0.001 and OR 0.70, p = 0.012.

**Table 2:** Logistic Regression and Odds Ratios for Cigarette Smoking Initiation (n = 88)

Variable	OR	95% CI	p-value
Gender (Male)	1.023	0.413, 2.533	0.961
Race (Non-white)	0.468	0.215, 1.018	0.056
Neuroticism	1.015	0.944, 1.092	0.690
Extraversion	0.984	0.921, 1.053	0.647
Openness	1.034	0.964, 1.110	0.349
Agreeableness	0.956	0.886, 1.032	0.251
Conscientiousness	0.871	0.802, 0.946	0.001
Sense of Coherence	1.010	0.977, 1.044	0.555
Self-efficacy	0.702	0.532, 0.927	0.012
Family support	1.012	1.000, 1.025	0.053
Friend support	0.999	0.991, 1.007	0.786

Note: OR = odds ratio; CI = confidence interval

### Smoking Frequency and Quantity

Self-efficacy emerged as the single most important predictor of frequency, F(11,37) = 2.77, p = <0.016, and quantity of cigarette smoking, F(11,37) = 2.11, p = < 0.05 (see Table 3). Students who reported lower levels of self-efficacy reported smoking cigarettes more frequently. Similarly, students who reported lower self-efficacy reported smoking greater quantities of cigarettes at any given time. Other personality factors, sense of coherence, coping resources, and demographic factors did not show any significant results on cigarette-smoking frequency or quantity.

**Table 3:** Impact of Self-Efficacy on Cigarette Smoking Frequency and Quantity.

Smoking Behavior	Predictor	Coefficient	SE	t	p-value	95%CI
Frequency	Self-efficacy	-0.223	0.052	-4.33	<0.0001	-0.329, -0.117
Quantity	Self-efficacy	-0.165	0.049	-3.36	0.002	-0.267, -0.064

## Discussion

Despite the clear evidence of the harmful effects of smoking, over half of the college students in this study reported that they had tried cigarettes. This number is alarming in that young people who experiment with cigarettes are more likely to become daily smokers in the future [51]. It was also noteworthy that the majority of the students in this study who reported smoking a whole cigarette did so during their adolescent years. These findings support previous findings regarding cigarette experimentation among adolescents [52] and indicate a clear need to target smoking prevention interventions to younger adolescents. At the same time, a substantial number of college students in this study began smoking at 17 years of age and older. Everett and Husten (1999) adeptly pointed out that although smoking initiation primarily occurs during adolescence, many young adults may also initiate their daily smoking patterns during college [53].

It is also reported that college-aged students have the most dramatic increase in cigarette smoking [4]. In this study the percentage of current cigarette smokers (23.7%) was slightly higher than what was reported in national surveys for adults (22.5%) and adolescents (22.9%) [54]. Although our sample of 161 college students may not represent all U.S. college students, the high prevalence of cigarette smoking among college students raises great concern. This concern is compounded by the fact that younger smokers (age 18 to 29) are less concerned about the negative health effects of smoking than older smokers (≥ 50 yeas of age) [5]. Similarly, Steptoe and colleagues (2002) found that the prevalence of smoking among European university students over a 10-year period increased regardless of increased health risk awareness. These findings suggest that college-aged students are particularly at risk worldwide for initiating as well as becoming daily cigarette smokers, alerting the increased need for setting up smoking prevention and cessation programs in colleges and universities [51], in addition to those programs at the K-12 school systems. Healthcare providers (physicians, nurses, pharmacists, oral health care providers, and psychologists, etc.) need to assume a key role in developing and implementing age-appropriate intervention programs to prevent tobacco addiction among the growing number of adolescent and college-aged smokers [52].

Previous research among adolescents has shown that smokers tend to be males [41] and Whites [52]. In our study, there was no significant difference in the number of males and females who had tried cigarette smoking. Ethnicity was also not a significant predictor of smoking behavior. In fact, contrary to previous research, gender and ethnicity were not significant predictors of smoking initiation, frequency, and quantity of cigarettes smoked per day among college students. These findings suggest that to be effective smoking-related intervention programs need to be targeted for both genders and all ethnic groups of college students in a more comprehensive manner.

Our approach was to simultaneously, rather than separately, examine the impact of personality factors (neuroticism, extraversion, openness, agreeableness, and conscientiousness), cognitive factors (sense of coherence, self-efficacy), coping resources (family and friend emotional social support) and demographic factors (gender and ethnicity) on cigarette smoking behaviors (initiation, frequency, and amount of cigarette smoking) among college students. Self-efficacy was identified as the single most significant predictor of initiation, frequency, and quantity of cigarette smoking. Self-efficacy is referred to as the individual's judgment of their capability to perform a specific task. In studies of health behaviors, self-efficacy has been noted to influence both an individual's choice of health behaviors and amount of effort dedicated to performing a specific behavior [55]. Self-efficacy also was found to be an important factor in preventing smoking initiation [2,6] and cigarette-smoking cessation among college aged individuals [5]. Consistent with these findings, we found that students who had higher levels of self-efficacy were less likely to try smoking cigarettes than those individuals with lower self-efficacy. Similarly, the students who reported higher levels of self-efficacy smoked less frequently and lower quantities of cigarettes than those with lower levels of self-efficacy. Thus, health care providers who develop smoking prevention and smoking cessation programs must concentrate on increasing self-efficacy among young adults to reduce the prevalence of cigarette smoking [33,45]. For example, Botvin and colleagues found that cognitive-behavioral intervention programs that incorporated personal self-management (overall self-efficacy, goal setting, and decision-making) along with generic social skills (assertiveness) and social resistance skills (confidence to avoid smoking) were more effective in preventing cigarette smoking, the effect of which lasted for at least six years [56].

Conscientiousness also was a significant predictor of cigarette smoking initiation. Students with higher levels of conscientiousness were less likely to try cigarette smoking than students with lower levels of conscientiousness. Individuals high in conscientiousness have been described as efficient, organized and goal-directed, while those with lower levels of conscientiousness are considered more impulsive and easier to persuade [10]. Costa and McCrae (1992) further explained that the more conscientious an individual is, the more competent, dutiful, orderly, responsible and thorough an individual appears to be. Not surprisingly, conscientiousness has also been linked to educational achievement and particularly to the will to achieve. Conversely, individuals with lower levels of conscientiousness may lack direction and have lower grades. This notion seems to support the findings of previous studies in which adolescents with poor scholastic achievement were more likely to experiment with cigarette smoking [57,58]. Identification of and targeting students with lower levels of conscientiousness and presumably lower academic performance may be a key strategy to reducing tobacco initiation.

Previous findings in adolescents have indicated that family social support, such as parental support, is an important protective factor in reducing the initiation and use of cigarettes [29,30]. Contrary to these earlier findings in adolescents, family emotional social support did not significantly reduce cigarette-smoking behavior among college students in this study. There may be a number of reasons for this discrepancy. First, many college students may have moved away from home and are more autonomous in their decision-making with smoking. Second, the way family emotional social support was measured in our study to include parents, siblings, grandparents, aunts, uncles, and cousins may have diluted the potential impact of parental emotional social support on smoking behaviors among college students. Alternatively, smoking behaviors of college students may have been influenced by family factors other than social support, such as parental modeling of cigarette smoking and family attitudes toward smoking, which was not measured in our study. In a previous study family smoking behaviors and parental modeling of smoking were associated with increased smoking in adolescents [59]. Further research is warranted in these areas to better understand smoking behaviors among college students.

Researchers have previously found that adolescent peer relationships also contribute to cigarette smoking. In our study with college students, however, friend or peer emotional support did not significantly predict smoking behaviors. In Kubus' (2003) review of the literature on peers and adolescent smoking, the author suggests that this relationship may not be as simple or overt as once thought. It is possible that, by the time students reach college ages, they may be less amenable to peer influences when making a decision on health behaviors, such as smoking behavior. Rather, future research may need to explore the role of romantic relationships (which are more prevalent in this age group) and their impact on smoking behavior among college students [34].

Although the findings of this study provide important insight into college students' smoking behaviors, the limitations of the study include the fact that data were gathered using all self-report measures and collected only once during the semester. Thus, we must rely on accurate reporting by the participants and no causal relationship between the predictor variables and smoking behaviors can be decisively determined. Prospective longitudinal investigations are needed to validate the causal relationship of personality factors, cognitive factors, and coping resources on smoking behavior among college students.

In summary, cigarette smoking contributes to over 440,000 deaths in the U.S. each year. Unfortunately, the prevalence of cigarette smoking continues to increase in the college-aged student regardless of the health risks associated with their use. The findings of our study support previous research that cigarette smoking is tried in adolescents but continues throughout the college years. Furthermore, low self-efficacy and the lack of conscientiousness were found to be determinants of smoking initiation while only low self-efficacy was a determinant of increased smoking frequency and quantity. The findings of our study suggest that strategies for smoking prevention and cessation intervention programs may need to be focused on increasing self-efficacy and conscientiousness to improve their success in college students. In this endeavor, health care providers may play a key role in developing and evaluating the effectiveness of smoking-related intervention programs.

## Competing interests

The authors declare that they have no competing interests.
